# Phenylketonuria and juvenile idiopathic arthritis: a case report

**DOI:** 10.1186/s12887-021-02602-6

**Published:** 2021-03-15

**Authors:** Ting Ting Zhu, Jin Wu, Li Yuan Wang, Xiao Mei Sun

**Affiliations:** 1 Department of Pediatrics, West China Second University Hospital, Sichuan University, 610041 Chengdu, Sichuan P. R. China; 2 Key Laboratory of Obstetric & Gynecologic and Pediatric Diseases and Birth Defects of Ministry of Education, Sichuan University, Chengdu, Sichuan China

**Keywords:** Phenylketonuria, Juvenile idiopathic Arthritis, Inflammation

## Abstract

**Background:**

Phenylketonuria (PKU) is a genetic metabolic disorder in which patients have no ability to convert phenylalanine to tyrosine. Several autoimmune diseases have been reported to combine with PKU, co-existent of PKU and Juvenile Idiopathic Arthritis (JIA) has not been presented.

**Case presentation:**

The girl was diagnosed with PKU at the age of 1 month confirmed by molecular data. At the age of 3.5 years, she presented with pain and swelling of her right ankle, right knee, and right hip joint. After a serial of examinations, she was diagnosed with JIA and treated with a nonsteroidal anti-inflammatory drug.

**Conclusions:**

We report a rare case of a 4-year-old girl with PKU and JIA, which supports a possible interaction between PKU and JIA. Long-term metabolic disturbance may increase the susceptibility to JIA. Further chronic inflammation could alter the metabolism of tryptophan and tyrosine to increase blood Phe concentration. In addition, corticosteroid and methotrexate therapy for JIA may increase blood Phe concentration.

## Background

Phenylketonuria (PKU) is a rare, genetic disease caused by mutations in the phenylalanine hydroxylase (PAH) gene; as PAH converts phenylalanine (Phe) to tyrosine (Tyr), its lack causes an accumulation of Phe [1]. Hyperphenylalaninemia will damage brain development and lead to significant intellectual impairment and behavioral disturbance. A Phe-restricted diet has improved the outcomes for patients with PKU. Arthritis has not been reported in patients with PKU except for those adult patients treated with pegvaliase therapy which has common adverse event of arthralgia. This is the first observed case of juvenile idiopathic arthritis (JIA) in a 4-year-old girl with PKU.

## Case presentation

A 4-year-old girl was considered as having phenylketonuria (PKU) at the age of 1 month due to her less dark pigmented hair and a positive neonatal screening for Phe.

**Fig. 1:**
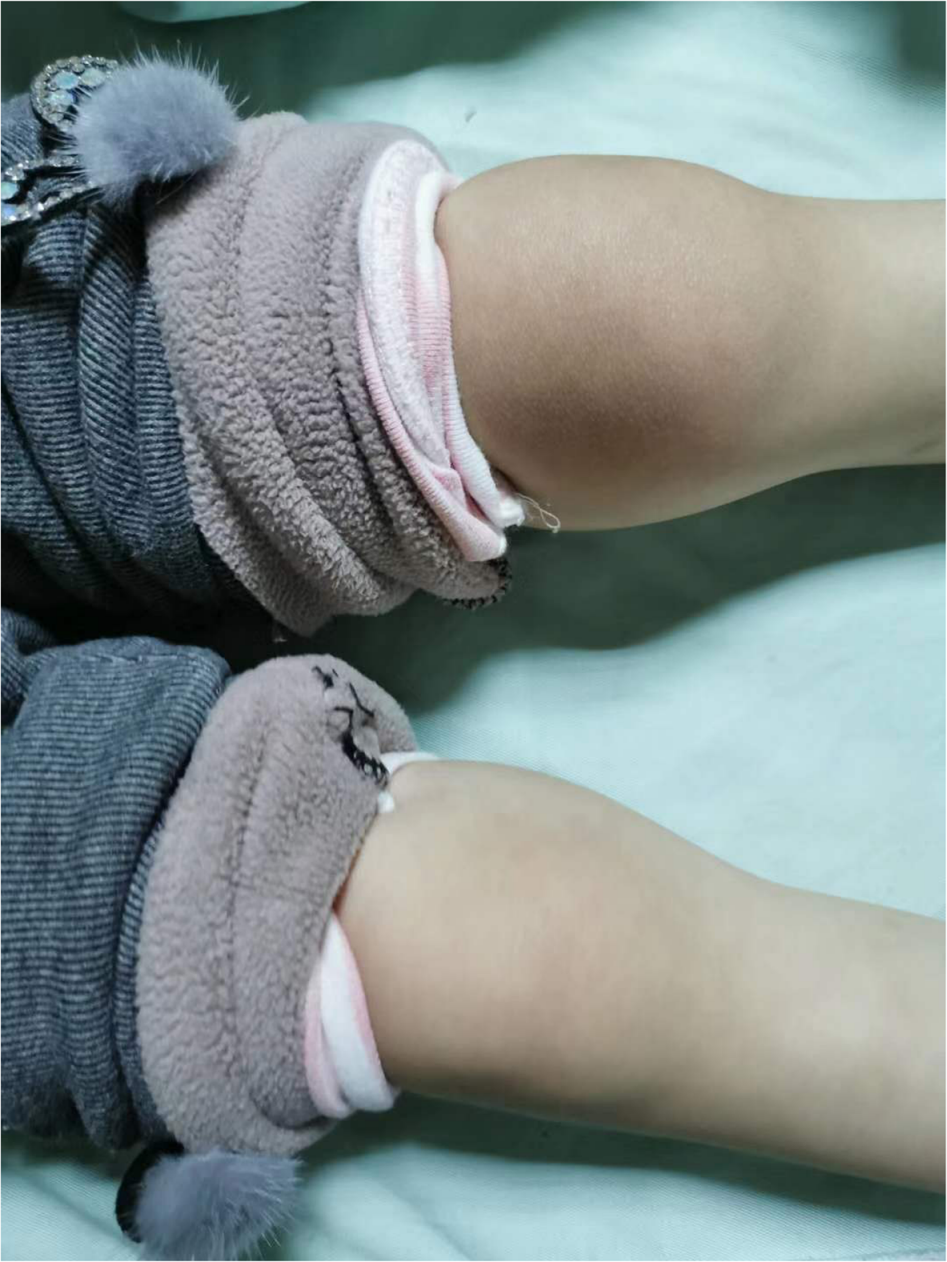
Arthritis of the knee joint

Her condition was confirmed by detecting homozygous mutation of *PAH* at nucleotide c.331 in exon 3 (c.331T > C) in the patient and heterozygous in both parents. The genetic sequencing was made in Beijing Kangxu Medical Research Center, Haidian District, Beijing, China. She was a term infant with a birth weight of 2700 g. There was no family history of arthritis or PKU. The baby was given a Phe-restricted diet. L-Amino acid-based medical foods (without Phe) provide ~ 80 % of the protein needs. The proportion of modified low-protein food was adjusted according to the concentration of regularly monitored blood Phe. She presented with normal motor development including walking and running, slight language delay and intellectual disability at 2 years old. At the age of 3.5 years, she presented with pain and swelling of her right ankle, right knee, and right hip joint (see Fig. 1). At that time, serum Phe concentration was 22.8 mg/dL. Unresolved ankle pain and elevated serum Phe concentration prompted referral to West China Second University Hospital of Sichuan University at the age of 4.5 years. She had severe pain of bilateral ankles, knees, and knuckles. She could not walk or jump. The affected joints were swollen, hot, and painful. A radiograph of the lower limbs showed bone demineralization. Laboratory investigation demonstrated an increase in Phe (19.52 mg/dL reference range: < 1.8 mg/dL), C-reactive protein (33.6 mg/L; reference range: 0-5 mg/L), erythrocyte sedimentation rate (36mm/h; reference range: < 21mm/h), tumor necrosis factor alpha (10.9pg/ml; reference range: <8.1pg/ml), and interleukin 6 (41.87pg/ml; reference range: < 5.9pg/ml), and a positive rheumatoid factor. Liver function, renal function, bone marrow biopsy smear and bone marrow culture were normal. Autoantibodies, antineutrophil cytoplasmic antibodies, anticardiolipin antibody, mycoplasma pneumoniae antibody, HLA-B27, and PPD tests were all negative. She was diagnosed with JIA and treated with a nonsteroidal anti-inflammatory drug (naproxen), methotrexate and low dose prednisone. Her joint pain responded well to the therapy. The patient has had followed-up appointments every 3 months for 1 year. Now she is thriving and can walk normally, with no further complaint of joint pain. Serum Phe concentration has been maintained within the high-normal range. (The last serum Phe concentration is 12 mg/dL)

## Discussion and conclusion

PKU has been reported to co-existent with several immune disorders, including scleroderma, ulcerative colitis, Type 1 diabetes mellitus, autoimmune hepatitis Type 2, alopecia universalis, and Grave’s disease, but not JIA [2]. JIA is a chronic idiopathic inflammatory disorder primarily involving joints. The peak incidence of JIA has been reported to occur at one to three years of age, with a preponderance of girls [3]. The underlying mechanism of JIA is not fully understood. Interactions among genetic factors, immune mechanisms, and environmental exposures are thought to contribute. The pathophysiology of PKU co-existence with JIA is speculated as follows. Firstly, given the complexity and heterogeneity of autoimmune disorders, metabolites have been explored to discover diagnostic or prognostic biomarkers for these diseases. The accumulation of some abnormal metabolites like glycosaminoglycans, adiponectin and leptin may be involved in the development and progression of joint dysfunction in JIA [4]. A recent metabolomics analysis revealed significantly higher ratios of both kynurenine/ tryptophan and phe/tyr and lower tryptophan levels in serum sample of JIA patients with high disease activity than those of clinically inactive patients [5]. The researches proposed a hypothesis that chronic inflammation could alter tryptophan and tyrosine metabolism [6]. Thus, PKU patients with chronic elevated Phe may be susceptible to JIA, in turn, co-existent JIA may have an effect on blood Phe. Finally, corticosteroid and methotrexate therapy for JIA may increase blood Phe concentration. MacDonald et al. reported that using corticosteroid was associated with increased blood Phe in 3/6 cases [2]. In our study, Phe concentration had decreased continuously since the onset of treatment for JIA. A much-restricted diet may play a role in decreasing Phe concentration. Thus, the influence of corticosteroid therapy in patients with PKU needs more studies to confirm. We will continue to follow up the patient’s response to steroids.

If a patient with PKU develops arthralgia, a diagnosis of JIA should be considered. This is the first reported case of a girl with PKU co-existent with JIA. Continued follow up of this girl will help us gain further knowledge on treating this rare comorbidity. The pathophysiology of PKU co-existence with JIA needs to be further explored.

## Data Availability

All relevant data are included in this manuscript and associated figures. However, if more information is required, the datasets analysed for the current study available from the corresponding author on reasonable request.

## Abbreviations

PKU: Phenylketonuria
JIA: Juvenile Idiopathic Arthritis
Phe: Phenylalanine
PAH: Phenylalanine hydroxylase
Tyr: Tyrosine

## References

[1] Blau N, van Spronsen FJ, Levy HL (2010). Phenylketonuria Lancet.

[2] MacDonald A, Ahring K, Almeida MF (2015). The challenges of managing coexistent disorders with phenylketonuria:30 cases. Mol Genet Metab.

[3] Sullivan DB, Cassidy JT, Petty RE (1975). Pathogenic implications of age of onset in juvenile rheumatoid arthritis. Arthritis Rheum.

[4] Winsz-Szczotka K, Mencner K, Olczyk K (2016). Metabolism of glycosaminoglycans in the course of juvenile idiopathic arthritis. Postepy Hig Med Dosw (Online).

[5] Korte-Bouws GAH, Albers E, Voskamp M (2019). Suffering from Chronic Inflammation Have Increased Activity of Both IDO and GTP-CH1 Pathways But Decreased BH4 Efficacy: Implications for Well-Being, Including Fatigue, Cognitive Impairment, Anxiety, and Depression. Pharmaceuticals (Basel).

[6] Capuron L, Schroecksnadel S, Féart C (2011). Chronic low-grade inflammation in elderly persons is associated with altered tryptophan and tyrosine metabolism: Role in neuropsychiatric symptoms. Biol Psychiatry.

